# Supplementary material for: Identification of photoperiod-induced specific miRNAs in the adrenal glands of Sunite sheep (Ovis aries)
Source: Front Vet Sci. 2022 Jul 22;9:888207. doi: 10.3389/fvets.2022.888207 (PMC9354845; doi:10.3389/fvets.2022.888207)
Supplement: Supplementary file 1 [file Data_Sheet_1.ZIP › Supplementary file/miRDeep2 file.zip!/miRDeep2 file/Know_Structure/LP42A2/oar-mir-381.pdf]

miRBase precursor : oar-mir-381
Total read count : 1721
oar-miR-381-5p read count : 2
oar-miR-381-3p read count : 1719
remaining reads : 0

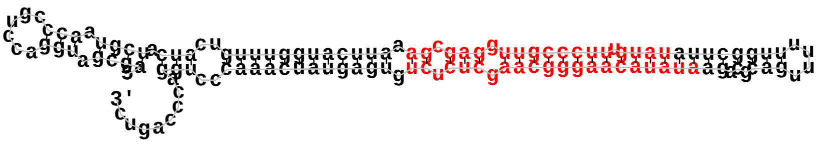

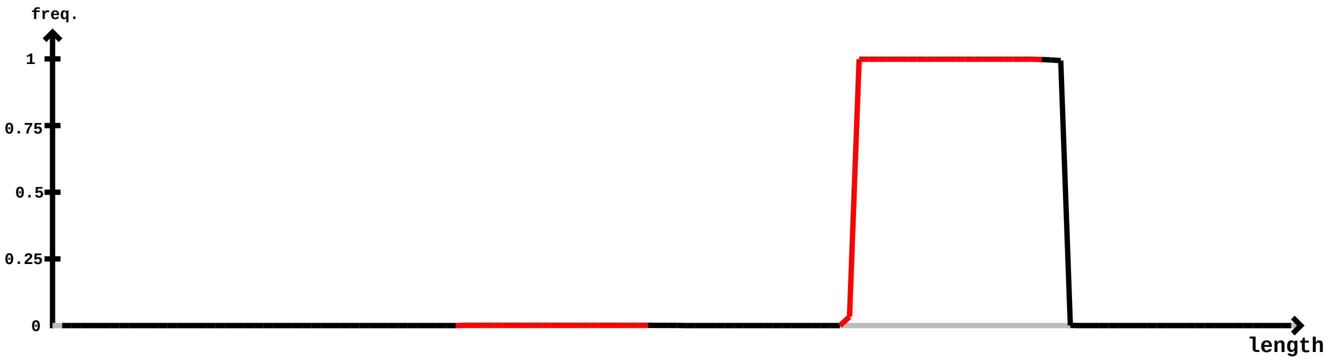


```
                                                                                 oar-miR-381-3p
                                         oar-miR-381-5p
5'- agcgauggaccugcccaaugcuacuacuguuugguacuuaaagcgagguugcccuuuguauauucgguuuuuugacgcagaauauacaagggcaagcucucugugaguaucaaacccuggacccaguc  -3'   exp
    ((((.(((......))).)))).(((..((((((((((((.((.(((.((((((((.((((((((((((....))).).)))))))))))))))).))).)).))))))))))))..)))........   reads     mm
    .........................................agcgagguugcccuuuguau...................................................................   1         0
    .........................................agcgagguugcccuuuguauauu................................................................   1         0
    .................................................................................auauacaagggcaagcucucugu........................   57        0
    ..................................................................................uauacaagggcaagcucuc...........................   2         0
    ..................................................................................uauacaagggcaagcucucu..........................   3         0
    ..................................................................................uauacaagggcaagcucucug.........................   4         0
    ..................................................................................uauacaagggcaagcucucugu........................   1652      0
    ..................................................................................uauacaagggcaagcucucugug.......................   1         0
```
